# Lycorine transfersomes modified with cell-penetrating peptides for topical treatment of cutaneous squamous cell carcinoma

**DOI:** 10.1186/s12951-023-01877-4

**Published:** 2023-04-28

**Authors:** Ying Li, Zongguang Tai, Jinyuan Ma, Fengze Miao, Rujuan Xin, Cuie Shen, Min Shen, Quangang Zhu, Zhongjian Chen

**Affiliations:** 1 Shanghai Skin Disease Hospital, School of Medicine, Tongji University, 1278 Baode Road, Shanghai, 200443 China; 2 Shanghai Engineering Research Center for Topical Chinese Medicine, 1278 Baode Road, Shanghai, 200443 China

**Keywords:** Lycorine, Cutaneous squamous cell carcinoma, Topical treatment, Transfersome, Cell-penetrating peptide

## Abstract

**Background:**

Topical anticancer drugs offer a potential therapeutic modality with high compliance for treating cutaneous squamous cell carcinoma (cSCC). However, the existing topical treatments for cSCC are associated with limited penetrating ability to achieve the desired outcome. Therefore, there remains an urgent requirement to develop drugs with efficient anticancer activity suitable for treating cSCC and to overcome the skin physiological barrier to improve the efficiency of drug delivery to the tumor.

**Results:**

We introduced lycorine (LR) into the topical treatment for cSCC and developed a cell-penetrating peptide (CPP)-modified cationic transfersome gel loaded with lycorine-oleic acid ionic complex (LR-OA) (LR@DTFs-CPP Gel) and investigated its topical therapeutic effects on cSCC. The anti-cSCC effects of LR and skin penetration of LR-OA transfersomes were confirmed. Simultaneously, cationic lipids and modification of R5H3 peptide of the transfersomes further enhanced the permeability of the skin and tumor as well as the effective delivery of LR to tumor cells.

**Conclusions:**

Topical treatment of cSCC-xenografted nude mice with LR@DTFs-CPP Gel showed effective anticancer properties with high safety. This novel formulation provides novel insights into the treatment and pathogenesis of cSCC.

**Supplementary Information:**

The online version contains supplementary material available at 10.1186/s12951-023-01877-4.

## Background

Cutaneous squamous cell carcinoma (cSCC) is the second most common skin cancer. The incidence of cSCC has been increasing worldwide, with a high risk of local recurrence, metastasis, and death in high-risk cSCC and advanced cSCC [1, 2]. In addition to surgical resection, other clinical treatment options include radiotherapy, conventional chemotherapy, biological therapy, and immunotherapy [1, 3, 4]. Topical treatment offers a potential therapeutic modality with high compliance for patients with skin cancer as it retains the drug locally in the tumor, minimizes systemic adverse effects, and is noninvasive [5, 6]. However, existing topical medication for cSCC, including topical fluorouracil and topical imiquimod, is only used for the local control of low-risk cSCC tumors owing to their inability to achieve deeper tumor penetration [7]. Nonetheless, these are not routinely applied to high-risk local cSCC or advanced cSCC because of their limited efficacy [8]. Therefore, drugs with efficient anticancer activity and transdermal delivery modalities that can effectively overcome physiological barriers, including the skin, tumor microenvironment, and cell barriers, are a promising alternative to exploit the advantages of cSCC-topical treatment and maximize the therapeutic effect and reduce adverse reactions.

Transdermal drug delivery is challenged by skin barriers, especially the stratum corneum, which is the outermost layer of the skin and the most important barrier to drug penetration [9]. Several means have been applied to enhance drug penetration through the skin, such as chemical penetration enhancers [10], iontophoresis [11], and electroporation [12]. In particular, nanoparticles, such as nanoemulsions [13], liposomes [14, 15], lipid nanoparticles [16, 17], and liquid crystals [18], are potential vehicles for delivering drugs through the skin because of their appropriate size, lipid composition with improved permeability, and easy surface modification [19]. Among them, transfersomes exhibit great potential in delivering anticancer drugs and improving skin penetration efficiency [20–23].

Transfersomes, also known as ultradeformable liposomes, are flexible or elastic liposomes containing phospholipids and surfactants. Compared with liposomes, transfersomes are highly deformable, allowing them to squeeze between keratinocytes and penetrate the skin efficiently, thus playing a vital role in promoting the transdermal penetration of their loaded drugs [24–26]. Negatively charged skin stratum corneum is conducive to electrostatic attraction of positively charged transfersomes. Hence, compared with negatively charged or electrically neutral transfersomes, positively charged counterparts can significantly enhance drug delivery through the skin [27–29]. The mechanism by which cations promote transdermal transfer may be related to their promotion of adsorption to and/or fusion with the stratum corneum [30]. Cell-penetrating peptides (CPPs) are a class of small peptides, cationic and/or amphiphilic, capable of penetrating cell membranes, cell layers, and tissues (including the skin), and they can carry macromolecules and small particles [31–35]. Among these, arginine oligomers are a type of CPP with significant permeation-promoting effects that exhibit strong affinity to anionic cell membranes due to the presence of multiple positively charged arginine, which is conducive to the internalization of the loaded drugs. The binding of cyclosporin A to arginine heptamer (R7) contributes to topical delivery and inhibition of inflammation [36], and its conjugation with the tetramer of arginine (R4) accelerates skin penetration of Gly-His-Lys for wound healing and skin care [37]. Arginine oligomers can further improve the skin and cell delivery of drug-loaded nanosystems [38, 39]. The CPPs comprising arginine and histidine can exert the penetrating effect of arginine and promote the endosomal escape of nanoparticles through the proton sponge effect of histidine, thus promoting the penetration of nanoparticles into the skin, which enhances the anti-tumor effect [40, 41]. In this study, we propose to modify transfersomes loaded with anticancer drugs by using CPP R5H3 to enhance transdermal and cellular penetration and thus improve anticancer effects.

**Fig. 1:**
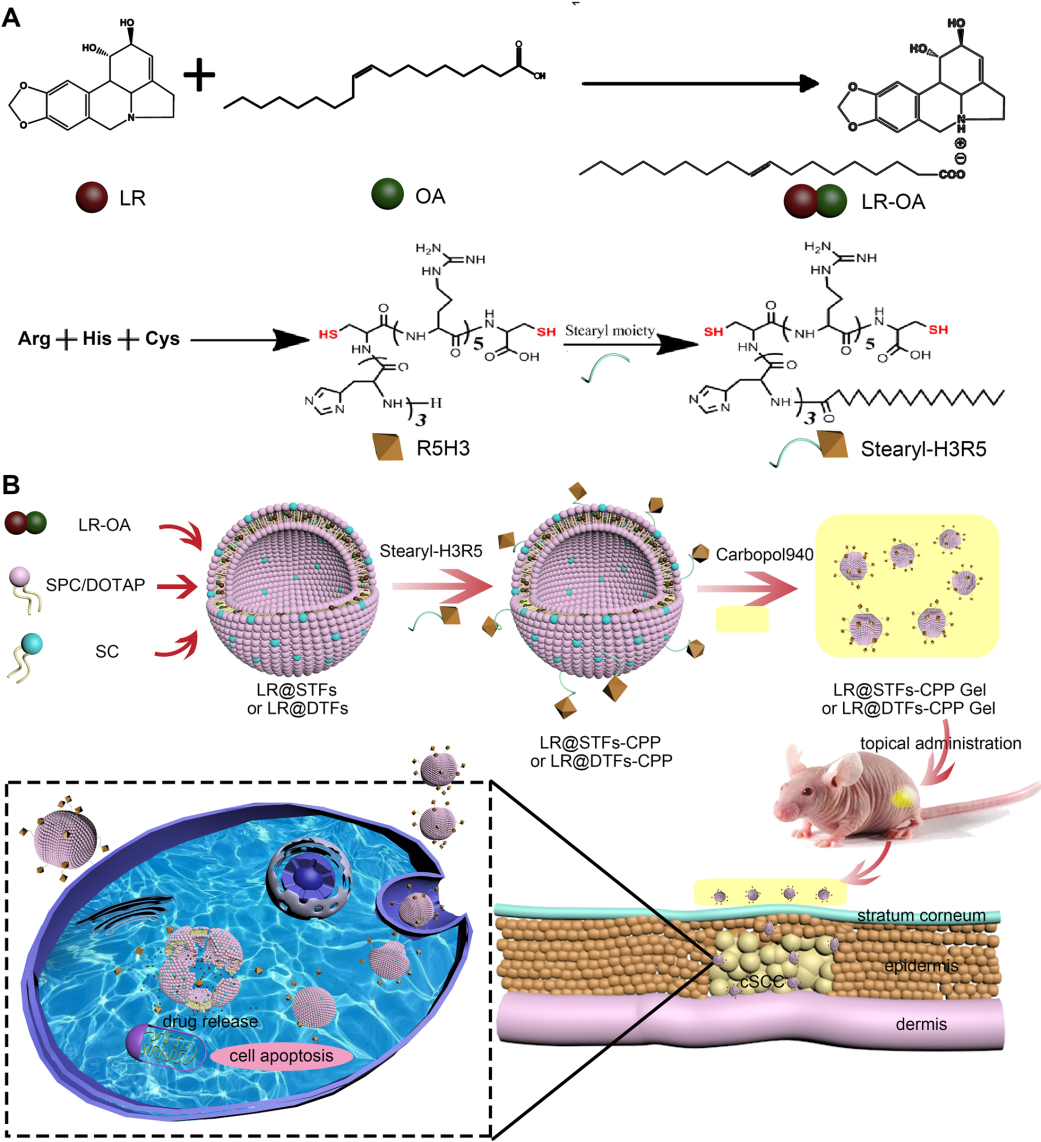
Schematic illustration of CPP-modified transfersome gel loaded with LR-OA for topical treatment of cSCC. **A** Preparation route of LR-OA and Stearyl-H3R5; **B** schematic representation of the preparation and application of LR@STFs-CPP Gel and LR@DTFs-CPP Gel as a paintable patch for topical drug delivery in noninvasive chemotherapy of cSCC resulting in improved transdermal efficiency of LR

In our efforts to develop improved anticancer drug formulations, plant sources have been found to provide bioactive compounds for developing new anticancer drugs. Lycorine (LR), an isoquinoline alkaloid (Fig. 1A), is extracted from the Amaryllidaceae plant family and displays a wide range of pharmacological properties, such as antiviral, antibacterial, anticancer, and anti-inflammatory effects [42]. LR exhibits potent anticancer activities in vitro at low concentrations against various cancers [43, 44], including drug-resistant cancer cells [45, 46]. LR has high specificity and is non-toxic to normal cells at concentrations that exert anticancer effects [47]. LR is effective against breast cancer [48], multiple myeloma [46], and ovarian cancer [49] in mice with xenograft tumors, with reduced toxicity. Its anticancer mechanisms include apoptosis induction, necrotic cell death, cell cycle and invasion inhibition, and metastasis [47]. These characteristics qualify LR as a potential anticancer drug of choice. However, owing to its poor solubility, the administration of free forms of LR does not correspond to good therapeutic outcomes. Furthermore, it is unsuitable to be included in nanocarriers, such as nanoparticles, liposomes, and nanoemulsions, to enhance its therapeutic effects because of its complex distribution in lipid matrices. This significantly limits the development of LR in anticancer drugs. Guo et al. prepared lycorine-oleic acid ionic complex (LR-OA) using the ion-pair formation method, shielding the charge of LR with the oppositely charged OA to enhance its lipophilicity. The prepared mannosylated nanoemulsion formulations loaded with this complex improved cellular uptake and cell growth inhibitory activity [50]. Zhao et al. also enhanced the liposolubility of the drug by forming an ion-pair complex of lycobetaine, a structural analog of LR, with OA, which improved the encapsulation of lycobetaine in nanoemulsion [51]. This method helped to facilitate the development of LR nanoformulations.

Owing to its potent anticancer effects and the lack of studies on its in vivo effects on cSCC, we anticipated that the topical application of LR to cSCC may provide a better therapeutic outcome. To this end, we developed LR nanoformulations to evaluate the topical application of LR against cSCC. After combining LR with OA, the drug-loaded nonionic and cationic transfersomes were prepared and modified with stearylated CPP Stearyl-H3R5 (Fig. 1A, Additional file 1). The stearyl acyl and CPP were connected by amide bonds, thus forming stable chemically modified structures. The in vitro anti-cell proliferation, cellular uptake, in vitro and in vivo transdermal effects, and in vivo animal efficacy and safety of LR and its nanoformulations were evaluated. Our study provides CPP-modified transfersome systems to load LR. The prepared systems can facilitate LR transdermal delivery and effective release into the tumor, thereby enhancing its safety and anticancer activity and providing a promising therapeutic strategy for the topical treatment of cSCC (Fig. 1B).

## Materials and methods

### Materials

DOTAP was purchased from Corden Pharma Switzerland LLC (Liestal, Switzerland). SPC was purchased from AVT Pharmaceutical Tech Co. Ltd. (Shanghai, China). LR and OA were purchased from Shanghai YuanYe Biotechnology Co. Ltd. (Shanghai, China). SC was purchased from Alfa Aesar Chemical Co. Ltd. (Shanghai, China). Stearyl-R3H5 was synthesized by NAMICROBIO Co. Ltd. (Xi’an, China). Roswell Park Memorial Institute 1640 medium (RPMI-1640), fetal bovine serum (FBS), and phosphate-buffered saline (PBS) were purchased from Gibco Life Technologies (Gaithersburg, MD, USA). Annexin V-FITC Apoptosis Detection Kit was supplied by Beyotime Biotechnology Co. Ltd. (Shanghai, China). Cell Counting Kit-8 was purchased from Dalian Meilun Biotechnology Co. Ltd. (Dalian, China). C6 and DAPI were acquired from J&K Scientific Ltd (Beijing, China). All other reagents were of analytical grade or better.

### Cells and animals

The human skin squamous cell carcinoma cell line (SCL-1) was obtained from Shanghai EK-Bioscience Biotechnology Co. Ltd. (Shanghai, China). The cells were cultured in RPMI 1640 medium containing 10% FBS, 100 μg/mL streptomycin, and 100 U/mL penicillin in a 5% CO_2_ humidified incubator at 37 °C.

The nude mice (BALB/c-nu, female, 4–5 weeks old) were provided by Shanghai JieSiJie Laboratory Animal Co. Ltd. All animal experiments were approved by the Ethics Committee of Shanghai Skin Disease Hospital and performed in accordance with the Guidelines for Care and Use of Laboratory Animals of Tongji University. SCL-1 cells (6 × 10^6^ cells) were injected subcutaneously into the nude mice on the back of the right hind leg to establish the xenograft cSCC tumor mouse model.

### Preparation of transfersomes and CPP-modified transfersomes

LR-OA was prepared as previously reported with minor modifications [50]. Briefly, LR and OA (1:2, w/w) were dissolved in dry tetrahydrofuran, and the solution was continuously stirred at 50 °C for 2 h. The solvent was then removed under reduced pressure, and LR-OA was obtained.

Transfersomes and CPP-modified counterparts were prepared using the thin film dispersion method followed by extrusion [52, 53]. Transfersomes with components of SPC and SC (6:1, w/w) (STFs) and CPP-modified STFs (STFs-CPP) with components of SPC, SC, and Stearyl-R3H5 (6:1:0.14, w/w/w) were dissolved in a mixture of organic solvents of ethanol and dichloromethane (1:2, v/v) and mixed thoroughly. Transfersomes with components of DOTAP and SC (6:1, w/w) (DTFs) and CPP-modified DTFs (DTFs-CPP) with components of DOTAP, SC, and Stearyl-R3H5 (6:1:0.14, w/w/w) were dissolved in ethanol and mixed thoroughly. LR-OA (the weight of LR accounted for 7.14% of the total lipid weight) was added into the components for the preparation of LR@STFs, LR@DTFs, LR@STFs-CPP, or LR@DTFs-CPP. To prepare the fluorescently labeled transfersomes, C6 (0.36% of the total lipid weight) was added. The organic solvent was removed under vacuum using a rotary evaporator at 40 °C until a dry film was formed. The film was then hydrated in deionized water for 30 min. The suspension obtained was sonicated using an ultrasonic cell crusher (Ningbo Xinzhi Biotechnology Co. Ltd., China) in an ice-water bath for 2 min (150 W for 1 s, interval 1 s) and extruded through polycarbonate membrane filters (Millipore, Billerica, MA, USA) using a mini-extruder (Avestin, Ottawa, Canada): first 10 times through a 0.2 μm pore size filter and then three times through a 0.1 μm pore size filter to obtain the final formulation. Blank transfersomes were prepared using the same procedure but without adding LR-OA.

### Characterization of transfersomes and CPP-modified transfersomes

The particle size, PDI, and zeta potential of transfersomes and CPP-modified transfersomes were determined at 25 ℃ using particle size and zeta potential analyzer Nanotrac Wave II (Microtrac, Largo, FL, USA). The morphology of LR-loaded CPP-modified transfersomes was visualized using TEM (HT7700; Hitachi, Tokyo, Japan). The encapsulation efficiency (EE) of various transfersome formulations was determined by ultrafiltration centrifugation. Freshly prepared (0.4 mL) LR@STFs, LR@DTFs, LR@STFs-CPP, or LR@DTFs-CPP (LR 1 mg/mL) was added into the ultrafiltration centrifugal tube (100 kDa, Millipore) and centrifuged at 6000 × *g* for 15 min. The collected filtrate and original formulation were diluted in ethanol with 1% acetic acid. For the quantitative determination of LR, its fluorescence intensity (λ_ex_/λ_em_ = 285/370 nm) was measured using a microplate reader (SpectraMax M2; Molecular Devices, Sunnyvale, CA, USA). The EE of LR in different formulations was calculated according to the following equation:

$${\text{EE}}\left( \% \right) = \frac{{{\text{W}}_{{\text{t}}} - {\text{W}}_{{\text{f}}} }}{{{\text{W}}_{{\text{t}}} }} \times 100\%$$

where W_t_ is the quantity of LR in the unfiltered formulation, and W_f_ is the quantity of LR in the filtering medium.

### Cellular uptake study

For fluorescence microscope observation, SCL-1 cells (1 × 10^5^ cells per well) were seeded into 6-well plates and incubated at 37 °C for 24 h. The cells were then incubated with free C6, C6 transfersomes (C6@STFs and C6@DTFs), and CPP-modified C6 transfersomes (C6@STFs-CPP and C6@DTFs-CPP) at a concentration of 0.5 μg/mL and incubated at 37 °C for 1 h. The cells were then washed three times in PBS and fixed in 4% paraformaldehyde for 20 min. The cells were washed in PBS, followed by staining the cell nuclei using DAPI (2 μg/mL) at 37 °C for 15 min and again washed in PBS and observed under a fluorescence microscope (Axio Vert. A1; Zeiss, Jena, Germany).

The cellular uptake of transfersomes was determined using flow cytometry. Briefly, SCL-1 cells were seeded into 6-well plates at 1.5 × 10^5^ cells per well and incubated at 37 °C for 24 h. The medium was then replaced with fresh medium containing various formulations, including free C6, C6@STFs, C6@DTFs, C6@STFs-CPP, and C6@DTFs-CPP at a concentration of 0.5 μg/mL, and incubated at 37 °C for 1 h. The cells were then washed thrice in PBS, followed by dissociation using trypsin, centrifuged at 1500 × *g* for 5 min, and resuspended in 0.5 mL PBS. The fluorescence intensity of C6 in the treated cells was determined using a flow cytometer (Beckman Coulter, Brea, CA, USA).

### Cell proliferation assay

Cell Counting Kit-8 assay was performed to evaluate the antiproliferative effect of various LR formulations, including free LR, LR@STFs, LR@DTFs, LR@STFs-CPP, and LR@DTFs-CPP. LR stock solution was prepared by dissolving it in DMSO. SCL-1 cells (5 × 10^3^ cells per well) were seeded into 96-well plates and incubated at 37 °C for 24 h. The medium was removed, and the cells were washed in PBS. After diluting the original formulations in fresh medium, 100 μL of various formulations containing different LR concentrations were dispensed into each well and incubated at 37 °C for 24 or 48 h. Subsequently, the Cell Counting Kit-8 solution was added. Finally, absorbance at 450 nm was measured using a microplate reader. Cell viability was calculated using the following formula:

$${\text{Cell}}\,{\text{viability}}\left( \% \right) = \frac{{{\text{A}}_{{\text{t}}} - {\text{A}}_{{\text{b}}} }}{{{\text{A}}_{{\text{c}}} - {\text{A}}_{{\text{b}}} }} \times 100\% ,$$

where A_t_, A_c_, and A_b_ represent the absorbance of treated cells, control cells, and blank, respectively. Each assay was performed in quintuplicate.

### In vitro apoptosis assay

In vitro apoptosis using various LR formulations was studied. SCL-1 cells (1.5 × 10^5^ cells per well) were seeded into 6-well plates and incubated at 37 °C for 24 h. Then, the medium was removed, and the cells were washed in PBS and incubated with various LR formulations, including free LR, LR@STFs, LR@DTFs, LR@STFs-CPP, and LR@DTFs-CPP at a concentration of 2 μM for 24 h. A blank medium was used as a control. The cells were dissociated using 0.25% trypsin, centrifuged at 1000 × *g* for 5 min, and resuspended in PBS, followed by centrifugation at 1000 × *g* for 5 min. The cells were then resuspended in 500 µL of Binding Buffer, followed by the addition of 5 µL Annexin V-FITC and 10 µL PI, and mixed gently. After incubation in the dark for 15 min, the double-stained cells were analyzed employing flow cytometry.

### In vitro transdermal delivery study

In vitro skin penetration experiments were performed using various LR formulations utilizing the Franz diffusion cell system. The diffusion area of the diffusion cell was 1.77 cm^2^, and the volume of the medium in the receptor chamber was 6.5 mL. The separated nude mouse skin was rinsed with PBS and fixed between the donor and receptor chambers, with the skin stratum corneum facing upward. Air bubbles formed between the bottom of the skin and the receptor solution were carefully removed. Fluorescent dye (C6) was used to determine the extent of skin penetration in vitro. Various C6 (10 μg/mL) formulations (0.5 mL) were deposited on the skin surface of the donor chamber. The receptor chamber was filled with PBS and ethanol (3:1, v/v) and stirred continuously in a water bath at 37 ± 1 °C. After 4 h, the skin was collected, repeatedly washed in PBS, polymerized in 4% paraformaldehyde, stained with DAPI, and examined under a fluorescence microscope (Axio Vert. A1). For quantitative analysis of skin penetration in vitro, various LR (200 μg/mL) formulations (0.5 mL) were deposited on the skin surface of the donor chamber, and each test formulation was performed in triplicate. The receptor chamber was filled with a mixture of PBS and ethanol with 1% acetic acid (3:1, v/v) and stirred continuously in a water bath at 37 ± 1 °C. Then, 0.5 mL of the medium was sampled from the receptor chamber at 0.5, 1, 2, 4, 6, 8, 10, and 12 h, respectively, and replaced with an equal volume of fresh medium, and the fluorescence intensity of LR (λex/λem = 285/370 nm) in the sample medium was determined. The cumulative permeation of LR per unit area, Q_n_ (μg/cm^2^), was calculated using the following equation:

$${\text{Q}}_{{\text{n}}} = \frac{{{\text{C}}_{{\text{n}}} {\text{V}} + \sum\nolimits_{i = 1}^{n - 1} {{\text{C}}_{{\text{i}}} {\text{V}}_{{\text{i}}} } }}{{\text{A}}},$$

where C_n_ is the concentration of LR in the medium in the receptor chamber at the n sampling point (μg/mL), V is the volume of the medium in the receptor chamber (mL), C_i_ is the concentration of LR in the medium in the receptor chamber at the i-sampling point (μg/mL), V_i_ is the volume sampled at each time point (mL), and A is the diffusion area of the diffusion cell (cm^2^).

After 12 h, the fixed mouse skin was collected to determine the quantity of LR in the skin. The skin was washed repeatedly with PBS and ethanol with 1% acetic acid (1:1, v/v) to remove residual LR adhered to the skin. The skin was cut into small pieces and homogenized in PBS and ethanol with 1% acetic acid (1:1, v/v) and then sonicated for 1 h. After centrifugation at 8000 × *g* for 20 min, the fluorescence intensity of LR in the supernatant was measured. The amount of drug retention per unit area of the skin, Q_s_ (μg/cm^2^), was calculated.

### Preparation of LR-loaded CPP-modified transfersome gel

For topical administration in animals and enhanced retention time in the skin over cSCC, we applied the gel as a reservoir for LR-loaded CPP-modified transfersomes. The Carbopol 940-based gel matrix (1.4%, w/v) was prepared by dispersing Carbopol 940 in water, stirring continuously, and allowing it to swell for 24 h. LR-loaded CPP-modified transfersomes (1 mg/mL) were added to the above gel matrix at a ratio of 1:1 and gently stirred to mix well, followed by neutralization with triethanolamine to pH 6.5 for obtaining LR-loaded CPP-modified transfersome gels. For comparative studies, free LR Gel was prepared using the same procedure, but by adding free LR instead of LR-loaded CPP-modified transfersomes. C6 was added to observe in vivo skin and tumor penetration.

### In vivo skin and tumor penetration study

We investigated the penetration of the more effective in vitro LR-loaded transfersome formulation and its gel in the skin and tumor of mice compared with free LR and its gel. Briefly, 0.1 mL of free C6, C6@DTFs-CPP, free C6 Gel, C6@STFs-CPP Gel, and C6@DTFs-CPP Gel at a concentration of 25 μg/mL was applied on the skin directly above the tumor of cSCC-bearing nude mice twice a day for 2 d. Subsequently, the mice were sacrificed, and the skin and tumors were collected. Tissue sections were obtained by cutting vertically from the skin toward the middle of the tumor, and the nuclei were stained with DAPI. Tissue sections were observed using fluorescence microscopy to compare in vivo skin and tumor penetration between the groups.

### In vivo anticancer study

The anticancer efficacy of the LR formulation was evaluated in cSCC-bearing nude mice. After subcutaneous injection of SCL-1 cells in nude mice, the tumor volume (length × width^2^/2) was measured using a caliper (Deli Tool Co. Ltd., Ningbo, China). When the tumor volume reached approximately 0.1 cm^3^, 20 nude mice were randomly divided into treatment and control groups (n = 5). The treatment groups were subjected to free LR Gel, LR@STFs-CPP Gel, and LR@DTFs-CPP Gel treatments. The tumor was topically plastered with LR formulation (2.5 mg/kg) twice a day for 14 days, whereas blank gel was applied to the control group. The tumor volume and body weight of mice were measured every 2 days. After the last treatment, the mice were sacrificed, and the tumor tissues were removed and photographed. Histopathology of the tumor sections was examined using H&E staining. Apoptotic cells were also labeled by performing TUNEL staining on tumor sections. The stained tissue sections were observed using a microscope (Olympus IX53, Tokyo, Japan).

### Safety assessment

To assess the safety of topical treatment with various LR formulations, the drug was applied locally to the tumor of each group of nude mice twice a day for 14 days, and the skin over the tumor was collected for histological examination. The major organs, including the heart, liver, spleen, lungs, and kidneys, were collected, and tissue sections were prepared and examined using H&E staining. Images were obtained using a microscope (Olympus IX53) for histopathological evaluation. Moreover, blood samples were collected from mice treated topically with free LR, LR@STFs-CPP, and LR@DTFs-CPP for 14 days and centrifuged at 3000 × *g* for 15 min. In the serum obtained, the levels of AST, ALT, and ALP (liver function indexes) and those of blood CRE and BUN (kidney function indexes) were measured.

### Statistical analysis

Statistical analysis was performed using GraphPad Prism 7.0 software, and the data are expressed as the mean ± standard deviation. For statistical analysis, two groups were compared using Student's *t*-test, and multiple groups were analyzed using a one-way analysis of variance to explore the significance of differences between groups. A *p*-value < 0.05 indicated statistical significance.

## Results and discussion

### Preparation and characterization of LR formulations.

To improve LR solubility in the organic phase necessary for its subsequent preparation of nanoformulations, we used the ion-pair formation method [50, 51] and selected the biodegradable and low toxic physiological long carbon chain fatty acid, OA, as the oppositely charged ion to enhance the lipophilicity of LR. LR-OA was formed after the carboxylate ion of OA attacked the protonated nitrogen on LR (Fig. 1A). Considering that the basicity of LR and the acidity of OA are weak, which makes the formation of ion pairs complex, we chose tetrahydrofuran with a low dielectric constant (dielectric constant 7.58) as the solvent to provide sufficient solubility for LR, OA, and their complexes and promote the formation of ion pairs. Meanwhile, we increased the amount of OA to more than twice that of LR (2.04:1, mol/mol) to allow LR to react with OA and ensure the formation of LR-OA.

Various LR-loaded transfersome formulations were prepared via thin film dispersion and extrusion methods. The image of the prepared CPP-modified LR transfersomes with components of 1,2-dioleoyl-3-trimethylammonium-propane (DOTAP) and sodium cholate hydrate (SC) (LR@DTFs-CPP) is shown in Additional file 2. The average particle size, polydispersity index (PDI), and zeta potential of various LR-loaded transfersome formulations are listed in Table 1. The average particle sizes of LR-loaded transfersomes LR@STFs (STFs, transfersomes with components of soybean phospholipid [SPC] and SC) and LR@DTFs were 80.9 and 77.6 nm, respectively. Stearylated R5H3, comprising a cationic peptide segment containing pentaarginine and trihistidine and a stearyl chain, was modified on the surface of LR-loaded transfersomes by hydrophobic and electrostatic interactions to obtain LR@STFs-CPP and LR@DTFs-CPP. Compared with that of unmodified transfersomes, the particle size of CPP-modified transfersomes increased from 80.9 ± 3.6 nm for LR@STFs to 98.0 ± 4.5 nm for LR@STFs-CPP (*p* < 0.01) and from 77.6 ± 5.0 nm for LR@DTFs to 92.3 ± 2.7 nm for LR@DTFs-CPP (*p* < 0.05), suggesting that the transfersomes were surface modified by R5H3 peptide.

**Table 1:** Characterization of LR-loaded transfersome formulations

Formulation	Size (nm)	PDI	Zeta potential (mV)	EE (%)
LR@STFs	80.9 ± 3.6	0.151 ± 0.014	−20.9 ± 5.3	84.6 ± 2.5
LR@DTFs	77.6 ± 5.0	0.135 ± 0.017	+ 8.2 ± 3.4	86.0 ± 1.6
LR@STFs-CPP	98.0 ± 4.5 **	0.143 ± 0.022	+ 15.3 ± 2.3	89.5 ± 1.9
LR@DTFs-CPP	92.3 ± 2.7 #	0.128 ± 0.018	+ 38.4 ± 1.3	90.8 ± 1.2

*LR* lycorine, *STFs* transfersomes with components of soybean phospholipid and sodium cholate hydrate, *DTFs* transfersomes with components of 1,2-dioleoyl-3-trimethylammonium-propane and sodium cholate hydrate, *CPP* cell-penetrating peptide, *PDI* polydispersity index, *EE* encapsulation efficiency

**p < 0.01 when compared with LR@STFs

^#^*p* < 0.05 when compared with LR@DTFs

**Fig. 2:**
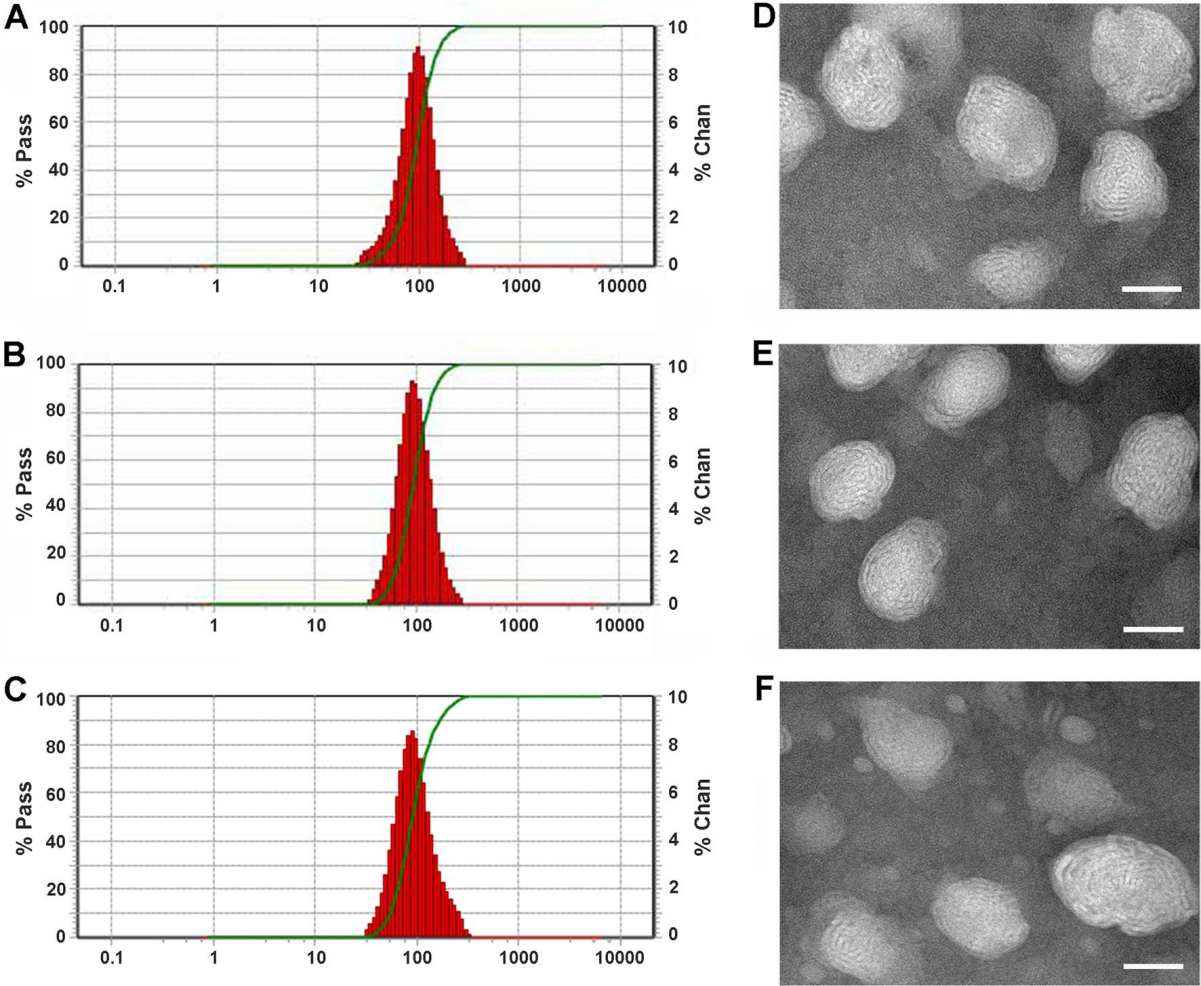
Characterization of LR@STFs-CPP. Particle size distribution of LR@STFs-CPP (**A**), LR@DTFs-CPP (**B**), and DTFs-CPP (**C**); TEM image of LR@STFs-CPP (**D**), LR@DTFs-CPP (**E**), and DTFs-CPP (**F**). Scale bar: 50 nm

As listed in Table 1, the PDI of all groups of LR-loaded transfersomes was less than 0.20, and the narrow PDI indicated that the prepared transfersomes had a uniform particle size distribution. Figure 2A, B and C showed that the CPP-modified transfersomes had a uniform particle size distribution. Transmission electron microscopy (TEM) images revealed the morphology of LR@STFs-CPP and LR@DTFs-CPP (Fig. 2D and E). From the particle size (Fig. 2C) and TEM (Fig. 2F) of blank DTFs-CPP, it could be seen that LR encapsulation had no effect on the structure of DTFs-CPP.

The zeta potential of LR@STFs was negative (− 20.9 mV; Table 1), which may have been caused by the anionic surfactant SC and excessive OA. In contrast, LR@DTFs had a positive zeta potential due to the cationic lipid DOTAP of phospholipid. The LR-loaded CPP-modified transfersomes LR@STFs-CPP and LR@DTFs-CPP had increased positive potentials (+ 15.3 and + 38.4 mV, respectively) based on LR@STFs and LR@DTFs due to the addition of cationic peptide fragments.

Owing to their poor solubility in lipid matrix, low lipophilic drugs are difficult to encapsulate with high efficiency in nanocarriers. In our study, the efficiency of various transfersome formulations encapsulating LR-OA was higher than 80% (Table 1), indicating that LR was efficiently loaded in the transfersomes. The formation of ion-pair complexes enhanced the lipophilicity of LR; thus, it was better solubilized in the lipid matrix, facilitating its high encapsulation levels into the transfersomes.

### Cellular uptake

**Fig. 3:**
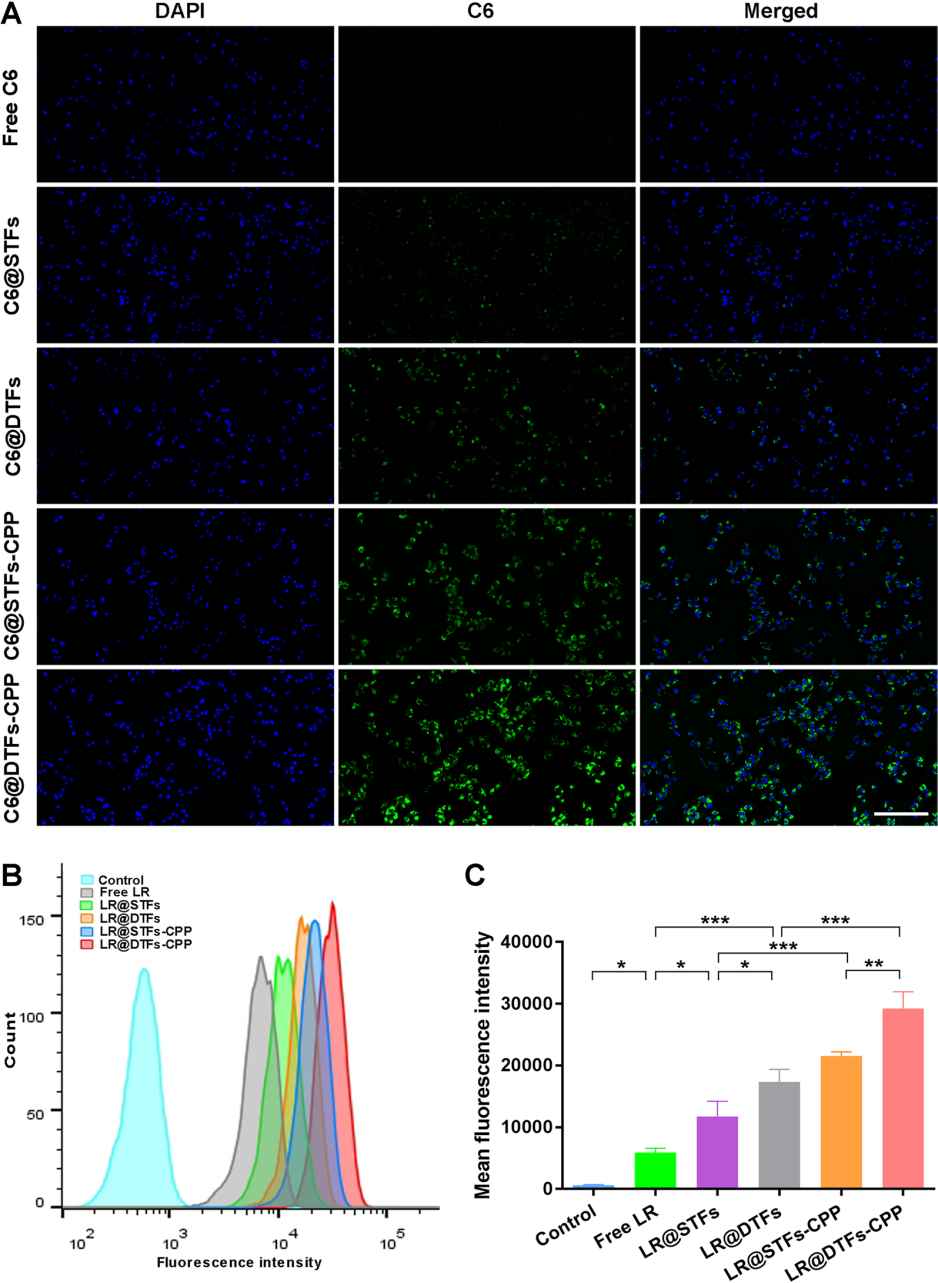
Cellular uptake of transfersomes on human skin squamous cell carcinoma (SCL-1) cells. **A** Fluorescence microscopy images of uptake of free C6, C6@STFs, C6@DTFs, C6@STFs-CPP, and C6@DTFs-CPP by SCL-1 cells. Scale bar: 500 µm; **B** quantitative analysis of the uptake of various C6 formulations using flow cytometry; **C** mean fluorescence intensity treated with various C6 formulations for 1 h; control represents untreated cells. Data are expressed as the mean ± SD (n = 3). *p < 0.05, **p < 0.01, and ***p < 0.001

The qualitative cellular uptake by human skin squamous cell carcinoma (SCL-1) cells when incubated with free Coumarin 6 (C6), C6@STFs, C6@DTFs, C6@STFs-CPP, and C6@DTFs-CPP for 1 h was performed using a fluorescence microscope. The C6 dye showed green fluorescence, and the nucleus showed blue fluorescence after 4,6-diamidino-2-phenylindole (DAPI) staining. The cellular uptake images of each group are shown in Fig. 3A. The cellular uptake of C6-loaded transfersomes prepared with the cationic phospholipid DOTAP was more intense than that of C6-loaded transfersomes prepared with SPC as a phospholipid, indicating that electrostatic interactions between the cationic surface and cell membrane may also mediate endocytosis. Furthermore, CPP-modified C6-loaded transfersomes exhibited stronger intracellular green fluorescence intensity than unmodified C6-loaded transfersomes, indicating that R5H3 peptide modification can promote cellular uptake of drug-loaded transfersomes.

To quantitatively study the effects of transfersomes and CPP-modified transfersomes on the uptake of encapsulated drugs, the cellular uptake of various C6 formulations was measured in SCL-1 cells using flow cytometry, with free C6 as a reference. The results showed that the intracellular fluorescence intensity was the lowest in SCL-1 cells treated with free C6 (Fig. 3B and C), indicating that the various transfersome formulations prepared could increase cellular uptake. Furthermore, the mean intracellular fluorescence intensity increased for C6@STFs-CPP compared with that for C6@STFs (*p* < 0.001) and for C6@DTFs-CPP compared with that for C6@DTFs (*p* < 0.001); it can also be deduced from Fig. 3B that SCL-1 cells treated with CPP-modified transfersomes showed a right shift in the flow cytometry analysis compared with non-CPP-modified transfersomes. This indicates that R5H3 peptide modification can promote cell membrane permeation of transfersomes, thereby increasing their cellular uptake. This result was consistent with that obtained from the qualitative cellular uptake study.

### Inhibition of cell proliferation

**Fig. 4:**
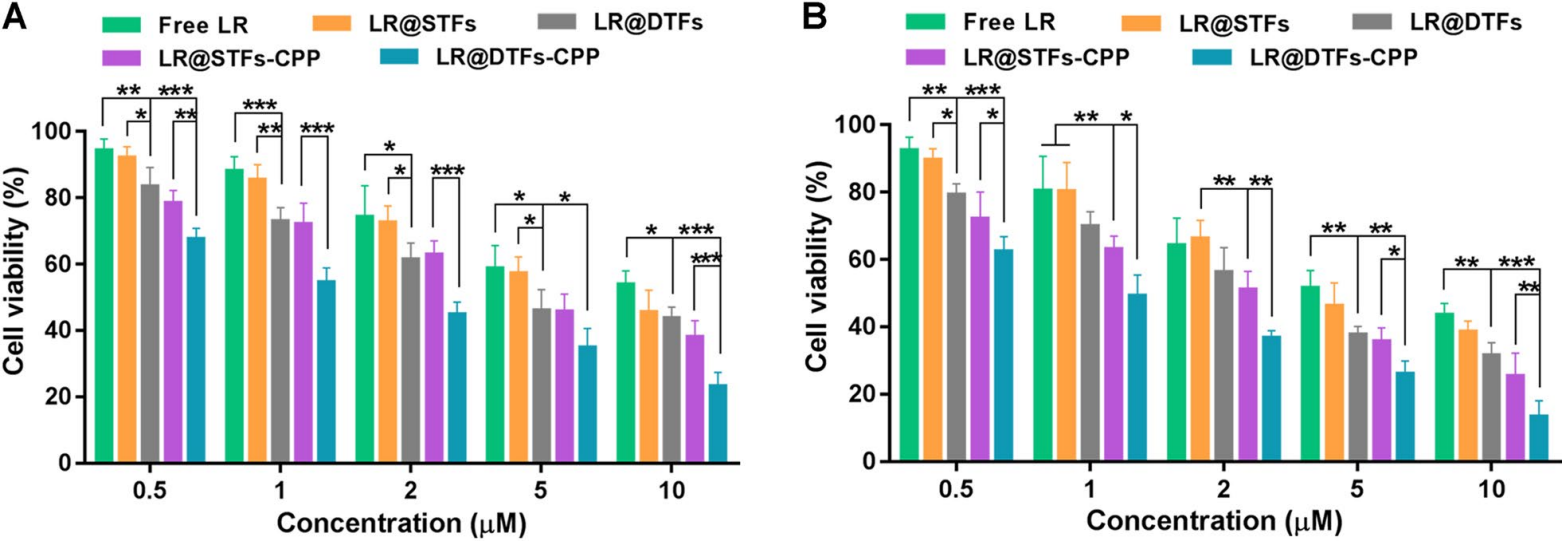
Cell proliferation inhibition was measured on human skin squamous cell carcinoma (SCL-1) cells. **A** Viability of SCL-1 cells treated with various LR formulations of different concentrations for 24 h; **B** viability of SCL-1 cells treated with various LR formulations of different concentrations for 48 h. *P < 0.05, **P < 0.01, and ***P < 0.001

SCL-1 cell proliferation inhibition by free LR, LR@STFs, LR@DTFs, LR@STFs-CPP, and LR@DTFs-CPP was detected using Cell Counting Kit-8 assay. The viability results of SCL-1 cells treated with various LR formulations of different concentrations for 24 and 48 h are shown in Fig. 4. Cell viability decreased with increasing LR concentrations (from 0.5 to 10 μM) in all treatment groups. The poor anti-proliferation effect for free LR might be attributed to its poor lipophilicity, limiting its cellular uptake. The cell proliferation inhibitory activity of LR@STFs and LR@DTFs obtained by preparing LR-OA to enhance the lipophilicity of LR and encapsulating them into transfersomes was higher than that of free LR. The half-maximal inhibitory concentration was lower for LR@STFs (4.90 μM) and LR@DTFs (2.97 μM) than that of free LR (5.95 μM) after treatment of SCL-1 cells for 48 h. This indicates that the LR-loaded transfersomes increased the intracellular concentration of the drug, resulting in more significant cancer cytotoxicity. The cell proliferation inhibitory activity was significantly lower for LR@STFs-CPP and LR@DTFs-CPP than that for LR@STFs and LR@DTFs, respectively, after treatment of SCL-1 cells for 24 and 48 h (Fig. 4A and B). The results indicate that the R5H3 peptide modified on the surface of transfersomes further facilitated the internalization of LR into SCL-1 cells, thereby enhancing their cellular uptake. These findings are consistent with those obtained from the fluorescence microscopy and flow cytometry cellular uptake experiments. Furthermore, cells treated with blank STFs-CPP and DTFs-CPP without LR for 48 h did not affect cell viability (Additional file 3), suggesting that the cell proliferation inhibitory effect of CPP-modified transfersomes on SCL-1 cells was mainly attributed to the LR anticancer activity rather than the toxicity of the nanocarriers.

### Apoptosis assay

**Fig. 5:**
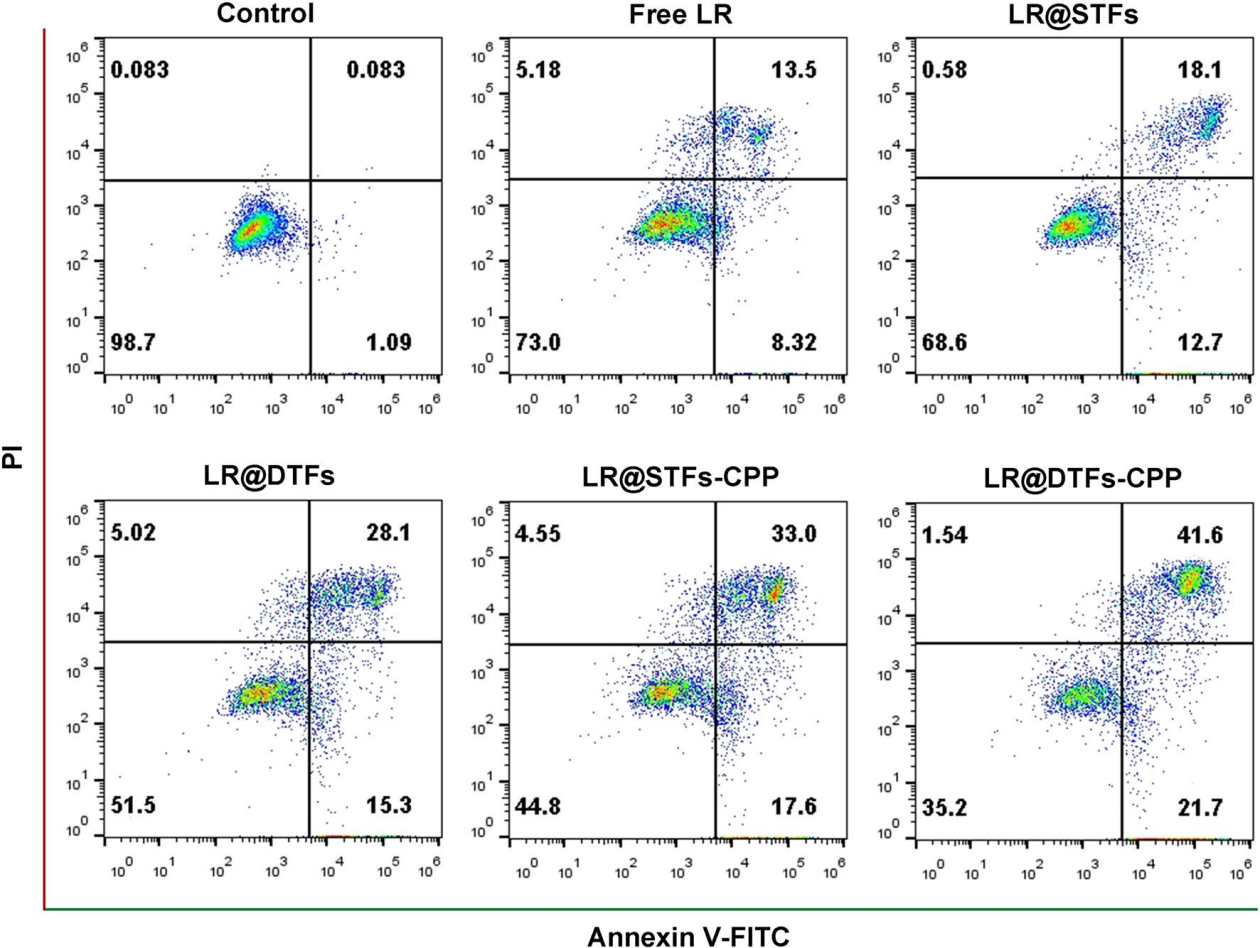
Evaluation of apoptosis in human skin squamous cell carcinoma (SCL-1) cells. SCL-1 cells were treated with various LR formulations at an LR concentration of 2 µM and analyzed using flow cytometry

Apoptosis is the most common anticancer mechanism for most anticancer therapies via death receptor-dependent exogenous or mitochondria-dependent endogenous pathways [54]. Apoptosis of SCL-1 cells was detected using Annexin V-FITC and propidium iodide (PI) double staining and flow cytometry analysis. The cells were incubated with free LR, LR@STFs, LR@DTFs, LR@STFs-CPP, and LR@DTFs-CPP, with each formulation containing 2 μM of LR. The effect of various LR formulations on the apoptosis of SCL-1 cells after 24 h is shown in Fig. 5. Compared with multiple LR-loaded transfersome formulations (LR@STFs, LR@DTFs, LR@STFs-CPP, and LR@DTFs-CPP), free LR resulted in the lowest apoptosis rate, including early apoptotic cells (lower right quadrant, Annexin V + , and PI −) and late apoptotic cells (upper right quadrant, Annexin V + , and PI +). CPP-modified transfersomes resulted in higher apoptosis than unmodified counterparts. Furthermore, LR@DTFs-CPP exhibited the highest effect on apoptosis. The results indicate that LR encapsulated as CPP-modified transfersomes has a superior anti-cSCC effect.

### In vitro skin penetration

**Fig. 6:**
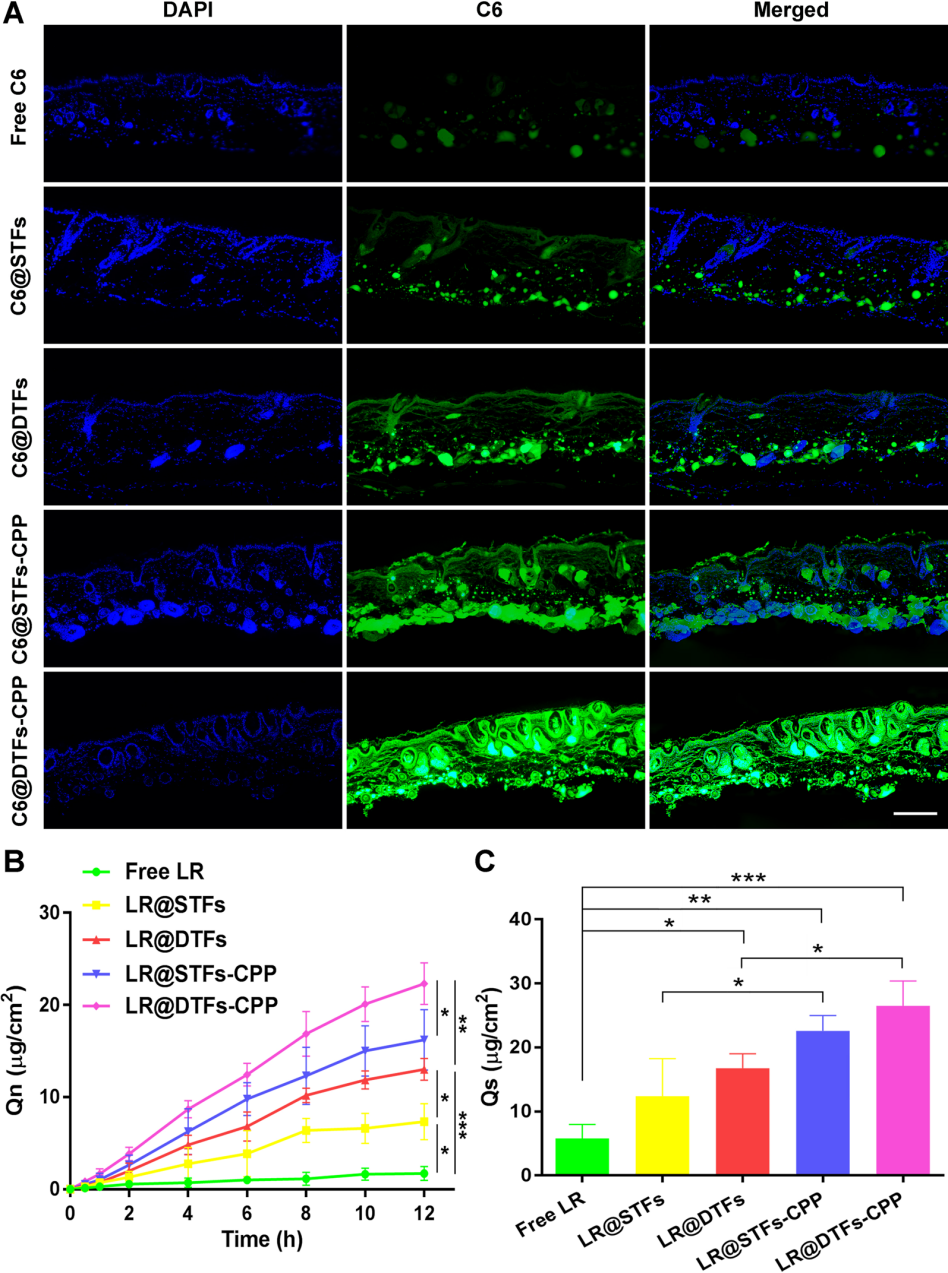
Transdermal properties of various LR formulations. **A** Fluorescence images of skin samples following incubation with various C6 formulations for 4 h. Scale bar: 400 μm; **B** cumulative transdermal amount of LR following incubation with various LR formulations; **C** retention of intradermal LR following incubation with various LR formulations for 12 h. *p < 0.05, **p < 0.01, and ***p < 0.001

The Franz diffusion cell system was used to assess the ability of each group of formulations to penetrate the skin separated from nude mice (Fig. 6). For the qualitative assay, the skin from each group of C6 formulation was collected following incubation in the Franz diffusion cell for 4 h. Fluorescence images of the skin are presented in Fig. 6A, which show that skin samples of the free C6 group exhibited only a weak C6 fluorescence signal following incubation for 4 h. In contrast, there were enhanced C6 fluorescence signals in the skin of all groups with C6-loaded transfersomes. Furthermore, more pronounced C6 fluorescence was observed in both the epidermis and dermis of C6@STFs-CPP and C6@DTFs-CPP groups, suggesting that R5H3 peptide could facilitate the penetration of the carrier into the deep layers of the skin.

For quantitative analysis of skin penetration in vitro, various LR (200 μg/mL) formulations (0.5 mL) were deposited on the skin surface of the donor chamber. The concentrations of LR were determined by sampling from the receptor chamber at 0.5, 1, 2, 4, 6, 8, 10, and 12 h. After calculating Q_n_, the LR transdermal penetration curve for each group was plotted (Fig. 6B). After sampling at 12 h, the cumulative permeation of LR per unit area (Q_8_) in the LR-loaded transfersome groups (LR@STFs and LR@DTFs) was 7.33 and 13.02 μg/cm^2^, respectively, both significantly (*p* < 0.05 and *p* < 0.001, respectively) higher than that in the free LR group (1.71 μg/cm^2^). This might be because the surfactants of transfersomes could change the crystal arrangement and increase the mobility of lipid molecules in the stratum corneum, thus enhancing the skin penetration of transfersomes. In addition, the high deformability of the transfersomes allowed them to squeeze between the stratum corneum and penetrate the skin, thus exhibiting enhanced penetration. Meanwhile, the Q_8_ of LR@DTFs was higher than that of LR@STFs (*p* < 0.05), indicating that the cationic transfersomes enhanced skin penetration. The Q_8_ of LR@DTFs-CPP was significantly increased compared with that of LR@DTFs (*p* < 0.01), which was the most potent skin penetration among various LR formulations because it combined the advantages of cationic transfersomes; R5H3 peptide modified on the surface of transfersomes further promoted transdermal penetration. The skin of nude mice with various LR formulations applied in each group was collected following incubation in a Franz diffusion cell for 12 h. Moreover, the Q_s_ for each group was calculated (Fig. 6C); compared with free LR, the LR-loaded transfersomes showed enhanced intradermal drug retention. In addition, the mean Q_s_ of LR@STFs-CPP and LR@DTFs-CPP groups were higher than that of LR@STFs and LR@DTFs groups, respectively (*p* < 0.05), indicating that the drug-loaded penetration into the skin of R5H3 peptide-modified transfersomes was significantly enhanced.

The above quantitative results of intradermal and transdermal penetration indicated that the drug-loaded transdermal penetration ability of the transfersomes was further enhanced by the transfersomes comprising cationic lipids and those modified by R5H3 peptide. These results were consistent with the qualitative findings observed via fluorescence microscopy.

### In vivo skin and tumor penetration

Our in vitro findings indicated that the anticancer effect and skin penetration of LR-loaded CPP-modified transfersomes were superior to those of the unmodified counterparts. Therefore, the former carriers were evaluated in further in vivo studies. To improve the retention capacity of CPP-modified transfersomes on the skin of cSCC-bearing nude mice, carbomer gel was applied as a reservoir for loading them. The image of the prepared LR@DTFs-CPP Gel is shown in Additional file 4.

**Fig. 7:**
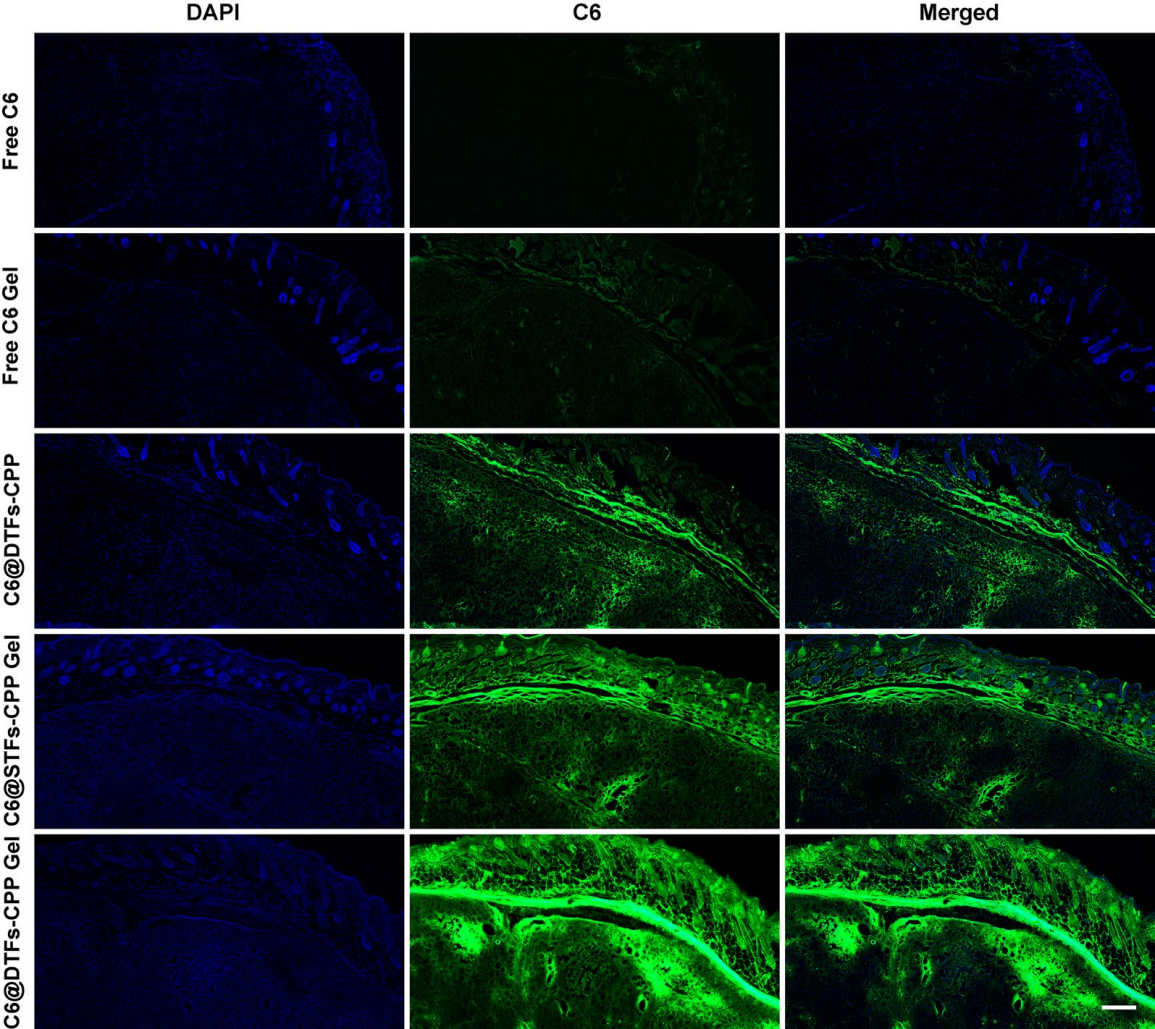
Fluorescence images of skin and tumor tissue sections from tumor-bearing nude mice. Mice were treated topically with free C6, C6@DTFs-CPP, free C6 Gel, C6@STFs-CPP Gel, and C6@DTFs-CPP Gel. Scale bar: 500 μm

After topical treatment of tumor-bearing nude mice with various C6 formulations, microdistribution of the preparations in the skin and tumor tissue was evaluated. Free C6, C6@DTFs-CPP, free C6 Gel, C6@STFs-CPP Gel, and C6@DTFs-CPP Gel were applied to the skin directly above the tumor twice a day. The mice were sacrificed after 2 days of treatment, the skin layer and subcutaneous tumor were collected, and tissue sections were prepared. The tissue sections of mice in each group were examined using fluorescence microscopy (Fig. 7). The C6 signals in the skin layer and tumor area after treatment with free C6 Gel and C6@DTFs-CPP Gel were more robust than those after treatment with free C6 and C6@DTFs-CPP. This indicated that incorporating transfersomes into the gel enhanced their transdermal delivery capacity after topical treatment as a patch on the skin directly above the tumor with prolonged retention time. The C6@STFs-CPP and C6@DTFs-CPP Gel showed significantly enhanced C6 signals in the skin layer and tumor area compared to free C6 Gel, indicating that the CPP-modified transfersome formulations promoted skin and tumor penetration in vivo.

As a CPP, the skin penetration mechanism of the R5H3 peptide is presumably associated with the transcellular and paracellular pathways. For transcellular pathways, the R5H3 peptide (rich in positively charged arginine) strongly interacts with phosphate groups on both sides of the membrane. This leads to distortion of the membrane structure, forming a pore leading to efficient transmembrane penetration of polyarginine peptide-conjugated nanoparticles [39]. In addition, histidine containing an imidazole group can improve intracellular delivery through its proton sponge effect on the endosomal escape of nanoparticles. For the paracellular pathway, the interaction between the R5H3 peptide and the skin extracellular lipid matrix leads to the destruction of ordered lipid orientation, thus generating channels for the transport of nanoparticles through the skin [40]. Meanwhile, the skin and tumor penetration of C6@DTFs-CPP Gel was more substantial than that of C6@STFs-CPP Gel. These results were consistent with those noted in the in vitro penetration experiments.

### In vivo anticancer efficacy and safety assessment

**Fig. 8:**
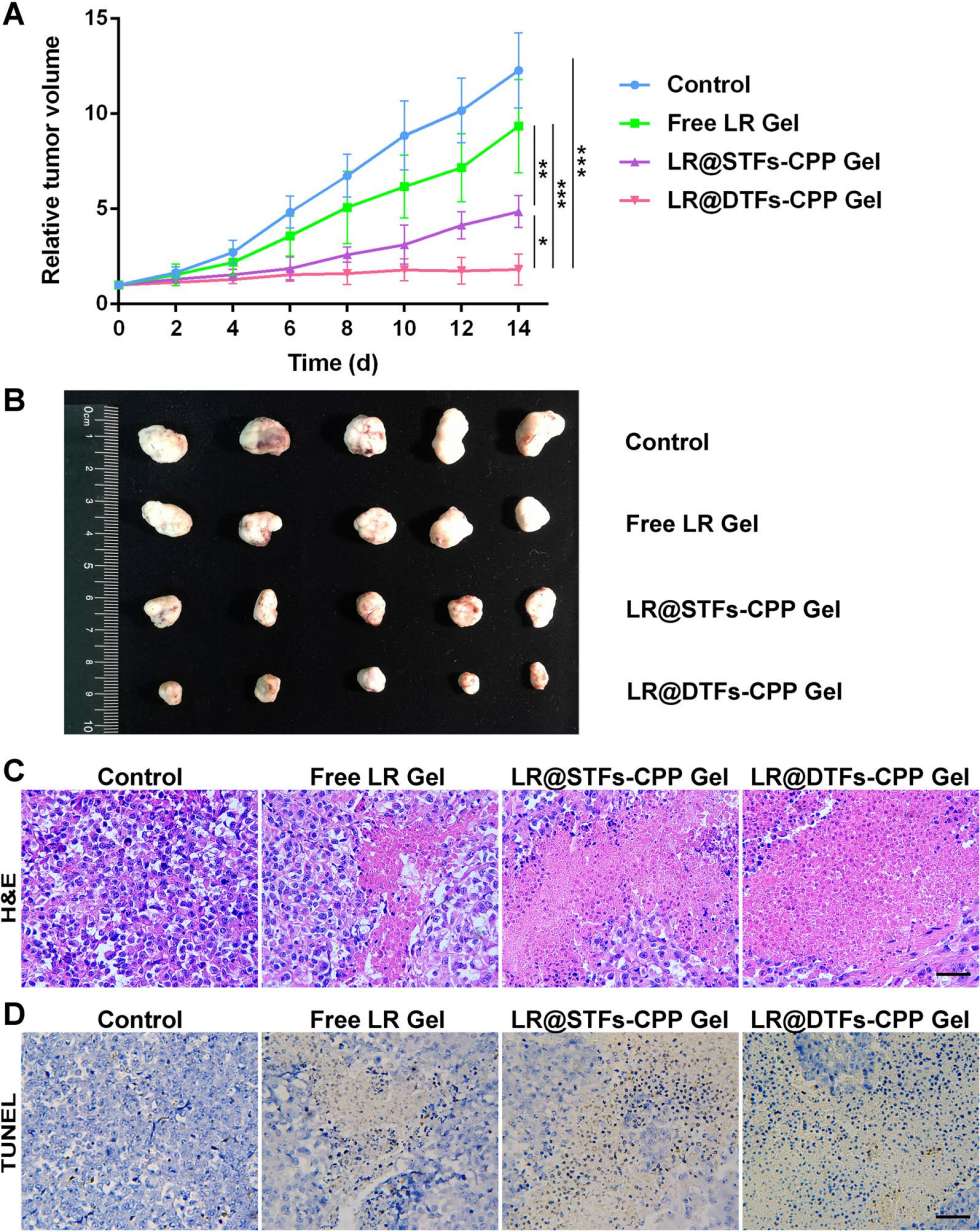
In vivo anticancer effects of transfersome gel formulations. Anticancer effects of LR and gel formulations were evaluated on the cutaneous squamous cell carcinoma (cSCC)-bearing nude mice model. **A** Tumor growth curves of control and treatment groups; **B** tumor images of each nude mouse group following 14 days of treatment; **C** histopathology of tumor sections stained with hematoxylin and eosin (H&E) following 14 days of treatment and **D** transferase-mediated dUTP nick end labeling (TUNEL)-stained images of tumor sections after 14 days of treatment. Scale bar: 50 μm. *p < 0.05, **p < 0.01, and ***p < 0.001

We evaluated the in vivo anticancer effects of various LR formulations applied topically for 14 days on the skin of cSCC-bearing nude mice. Additional file 5 includes representative images of each group throughout the 14-d topical treatment. Although the tumor size and weight in the free LR Gel group after 14 days of treatment was smaller than that in the control counterpart, there was no significant difference between the two groups (*p* > 0.05). This suggests that topical treatment with free LR Gel may lead to weak anti-tumor activity (Fig. 8A and B, Additional files 4 and 5). In contrast, the tumor growth in nude mice treated with LR-loaded CPP-modified transfersome gel formulation was significantly inhibited (*p* < 0.001), suggesting that R5H3 peptide-modified transfersomes could enhance the in vivo anticancer efficacy of LR. After 14 days of treatment of nude mice, the tumor growth of the LR@DTFs-CPP Gel group was consistently the lowest among the groups, indicating that LR@DTFs-CPP Gel had the strongest anticancer effect (Fig. 8A and B, Additional file 6). This was consistent with the in vitro anticancer activity experiments and in vivo skin and tumor penetration study.

The histopathological changes of the tumors in each group were examined at the end of the experiment. Hematoxylin and eosin (H&E) staining showed that free LR gel exhibited limited anticancer effects compared with the control group. The inhibitory activity of LR in the gel prepared after R5H3 peptide-modified transfersomes encapsulated it was more potent than that of the free drug (Fig. 8C). LR@DTFs-CPP Gel treatment resulted in a smaller tumor area than LR@STFs-CPP Gel treatment, further confirming that the enhanced positive charge on the surface of transfersomes could interact with the negatively charged skin surface to achieve better topical drug delivery (Fig. 8C, Additional file 7). Terminal deoxynucleotidyl transferase-mediated dUTP nick end labeling (TUNEL) staining of tumor tissue sections also revealed that CPP-modified transfersome gel, but not free LR gel, induced strong apoptotic activity; LR@DTFs-CPP Gel induced the highest level of apoptosis in tumor tissues (Fig. 8D). By applying transfersomes suitable for transdermal drug delivery, as nanocarriers for LR delivery and surface modification of transfersomes with CPPs, to enhance skin and tumor penetration, and combining the enhanced penetration of the cationic transfersomes and their affinity for tumor cells for topical application of the skin directly above the tumor, we improved the penetration ability of LR into the skin and tumor, effectively delivered LR into cSCC cells, and, thus, induced a potent anticancer effect. Therefore, our results suggest that topical application of LR@DTFs-CPP Gel can effectively treat cSCC.

**Fig. 9:**
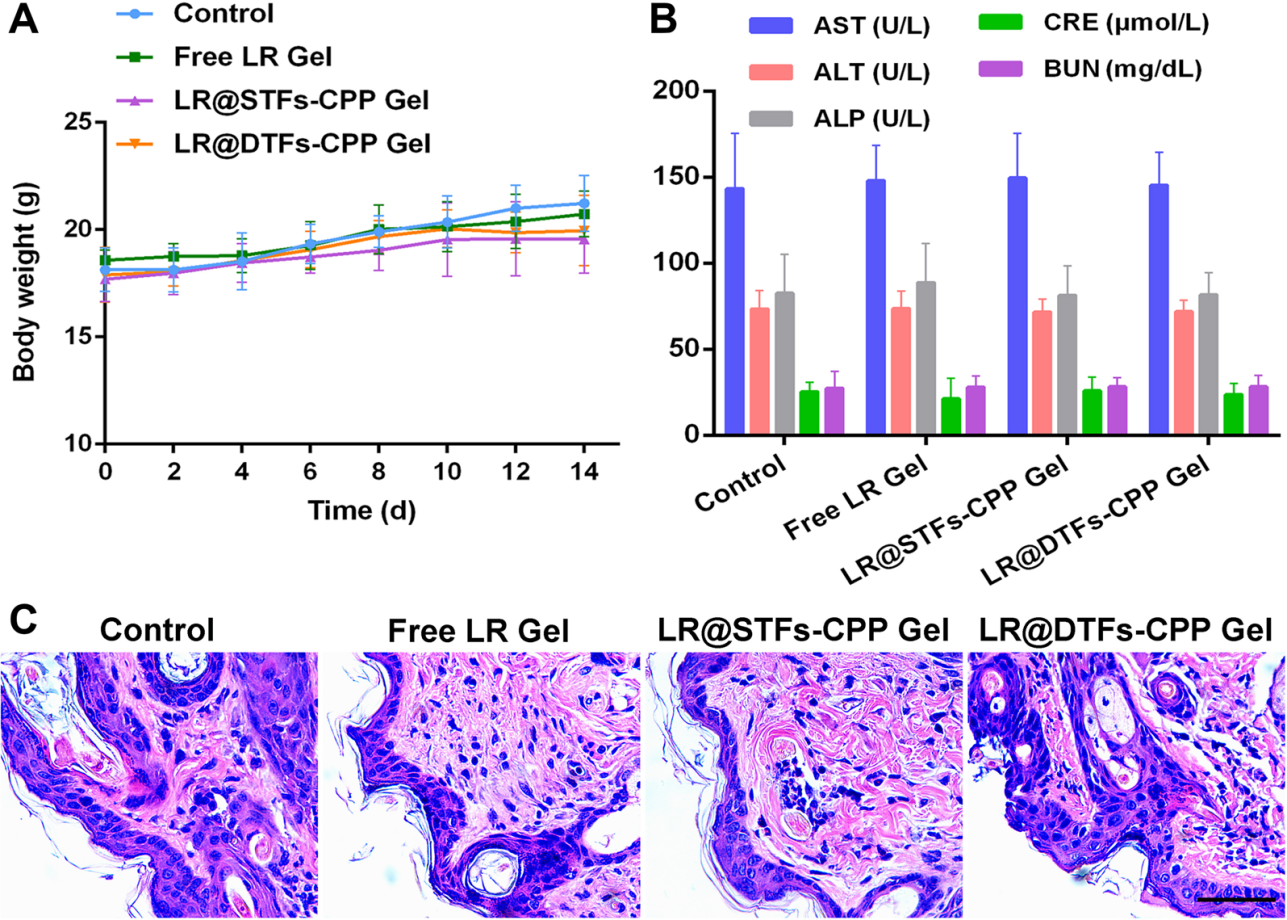
In vivo safety assessment of tumor-bearing nude mice. **A** Changes in body weight of mice post-treatment; **B** aspartate aminotransferase (AST), alanine aminotransferase (ALT), alkaline phosphatase (ALP), creatinine (CRE), and blood urea nitrogen (BUN) levels in nude mice from each group following 14 days of treatment; **C** histological assessment of drug-plastered skin of nude mice following 14 days of treatment with various formulations. Tissues were stained with hematoxylin and eosin. Scale bar: 50 μm

The body weight changes of tumor-bearing nude mice were monitored. The weight of the animals in all treated groups did not significantly decrease after 14 days of treatment (Fig. 9A), indicating that various LR formulations were tolerable. In addition, all treated mouse groups showed no significant histopathological damage in the H&E staining of sections from major organs (heart, liver, spleen, lung, and kidney) (Additional file 8).

Moreover, we collected the skin directly above the tumor after 14 days of topical application for histological evaluation (Fig. 9C) and observed no significant pathological changes in the skin tissues at the administration site in all groups. To further evaluate the effects on liver and kidney functions of mice after topical treatment with various LR formulations, blood samples were collected from mice in each group after 14 days of treatment. Changes in serum activity in aspartate aminotransferase (AST), alanine aminotransferase (ALT), alkaline phosphatase (ALP) (liver function indexes), creatinine (CRE), and blood urea nitrogen (BUN) (renal function indexes) were measured. No significant changes in liver and renal function indexes were observed in each LR treatment group compared with the control counterpart (Fig. 9B). This indicates that none of the LR formulations applied topically impaired the liver and renal functions of treated mice. The results signify the safe use of LR-loaded transfersome gel formulations developed in this study.

The results of the in vivo treatment studies confirmed the efficacy and safety of the LR-loaded R5H3 peptide-modified transfersome gel formulations in the topical treatment of cSCC-bearing nude mice. LR was first prepared into liposomes and then into hydrogels, which solved the disadvantage of poor dispersion of LR in hydrogel matrix. In addition, hydrogels ensure the slow release of LR, so the gel system avoids the side effects of LR on healthy tissue. The enhanced anticancer efficacy of LR@DTFs-CPP Gel may be attributed to the surface modification of CPP-modified transfersomes to enhance skin and tumor permeation, which enhances skin and tumor permeability. Meanwhile, cationic metastases enhanced the permeability and affinity of tumor cells. LR released locally is more likely to pass through the skin and infiltrate the tumor, thus achieving the combination of cSCC's penetration-promoting therapy.

## Conclusions

In this study, LR was introduced into the ex vivo evaluation study of cSCC. The solubility of LR was modified by preparing LR-OA ionic complex to enable its encapsulation into transfersomes for enhancing skin and tumor permeability for topical treatment of cSCC. The prepared LR-loaded transfersomes had a smaller particle size, smaller PDI, higher EE, and more significant transdermal penetration and anticancer effects than free LR. The addition of cationic lipids and modification of CPPs further enhanced the transdermal delivery, tumor penetration, and intracellular release. Our study found that LR@DTFs-CPP Gel topical chemotherapy showed effective anti-cSCC effects and high safety. This work provides novel insights into the application of LR in cSCC and the development of CPP-modified cationic transfersomes for topical use in the treatment of cSCC.

## Supplementary Information

**Additional file 1: Figure S1.** ^1^H-NMR (600 MHz) spectrum of R5H3 (A) and Stearyl-H3R5 (B).

**Additional file 2: Figure S2.** Representative image of cell-penetrating peptide modified lycorine transfersomes with components of 1,2-dioleoyl-3-trimethylammonium-propane and sodium cholate hydrate (LR@DTFs-CPP).

**Additional file 3: Figure S3.** Viability of human skin squamous cell carcinoma (SCL-1) cells incubated for 48 h with blank cell-penetrating peptide modified transfersomes with components of soybean phospholipid and sodium cholate hydrate (STFs-CPP) and cell-penetrating peptide modified transfersomes with components of 1,2-dioleoyl-3-trimethylammonium-propane and sodium cholate hydrate (DTFs-CPP) without lycorine (LR).

**Additional file 4: Figure S4.** Representative image of cell-penetrating peptide modified lycorine transfersomes with components of 1,2-dioleoyl-3-trimethylammonium-propane and sodium cholate hydrate (LR@DTFs-CPP) Gel.

**Additional file 5: Figure S5.** Representative images of cutaneous squamous cell carcinoma (cSCC) tumor-bearing nude mice treated with various formulations.

**Additional file 6: Figure S6.** Average tumor weight of mice in each group after 14 days of treatment. *p < 0.05, **p < 0.01, and ***p < 0.001.

**Additional file 7: Figure S7.** Histopathology of tissue sections of the skin with cutaneous squamous cell carcinoma (cSCC) tumor stained using hematoxylin and eosin after 14 days of treatment. Scale bar: 400 μm.

**Additional file 8: Figure S8.** Histological assessment of the major organs of nude mice treated with various formulations for 14 days. Tissues were stained using hematoxylin and eosin (H&E). Scale bar: 100 μm.

## Data Availability

The data are available from the corresponding author on reasonable request.

## Abbreviations

ALP: Alkaline phosphatase
ALT: Alanine aminotransferase
AST: Aspartate aminotransferase
BUN: Blood urea nitrogen
CPPs: Cell-penetrating peptides
CRE: Creatinine
cSCC: Cutaneous squamous cell carcinoma
DAPI: 4, 6-Diamidino-2-phenylindole
DTFs: Transfersomes with components of DOTAP and SC
DTFs-CPP: CPP-modified DTFs
EE: Encapsulation efficiency
FBS: Fetal bovine serum
H&E: Hematoxylin and eosin
LR: Lycorine
LR-OA: Lycorine-oleic acid ionic complex
PBS: Phosphate-buffered saline
PDI: Polydispersity index
PI: Propidium iodide
RPMI-1640: Roswell Park Memorial Institute 1640 medium
SC: Sodium cholate hydrate
SCL-1: Squamous cell carcinoma cell line
SPC: Soybean phospholipid
STFs: Transfersomes with components of SPC and SC
STFs-CPP: CPP-modified STFs
TEM: Transmission electron microscopy
TUNEL: Transferase-mediated dUTP nick end labeling
