# Supplementary figures and images for: Lycorine transfersomes modified with cell-penetrating peptides for topical treatment of cutaneous squamous cell carcinoma
Source: J Nanobiotechnology. 2023 Apr 28;21:139. doi: 10.1186/s12951-023-01877-4 (PMC10148442; doi:10.1186/s12951-023-01877-4)

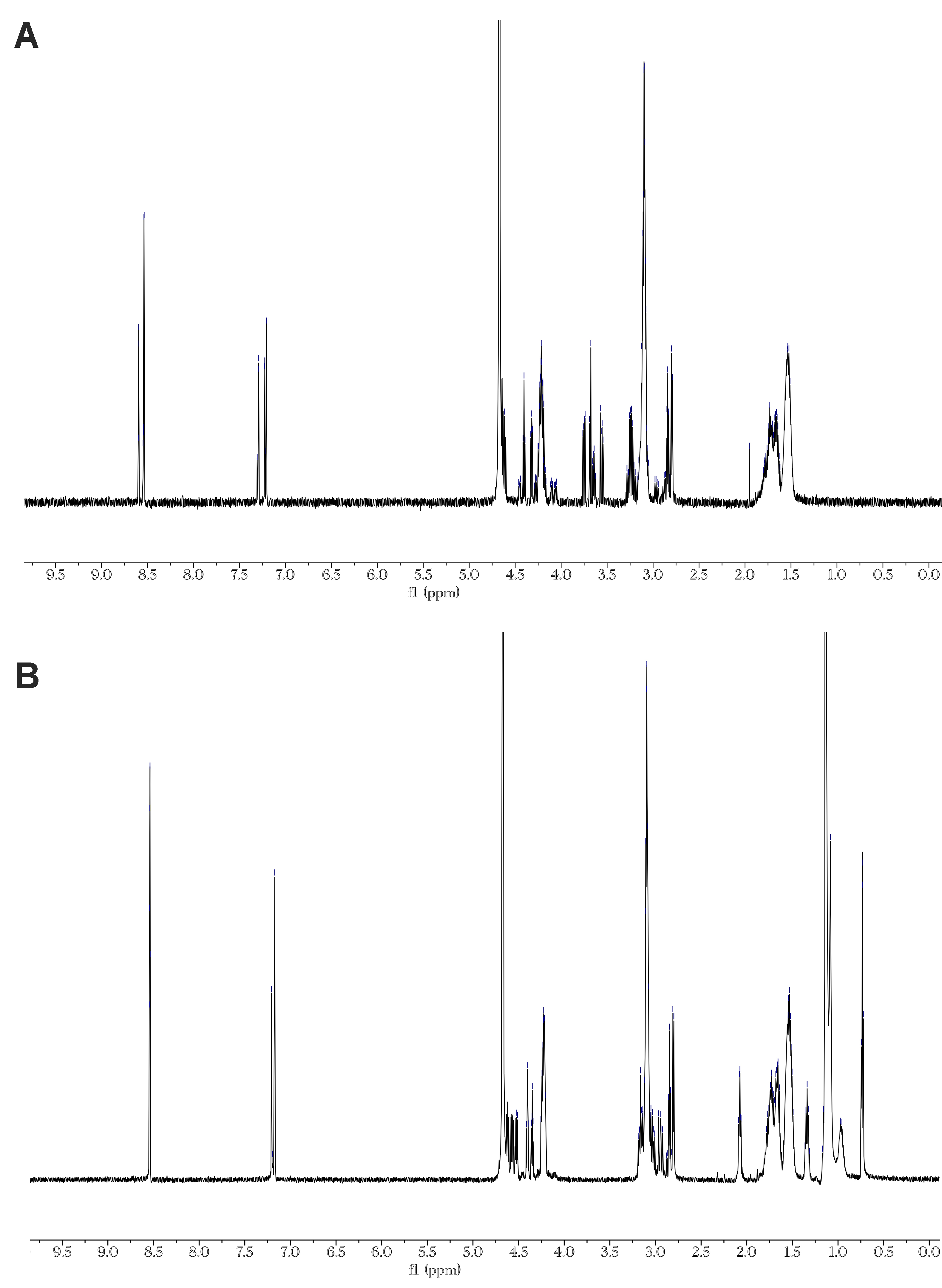

Supplement: Supplementary file 1 — Additional file 1: Figure S1. 1H-NMR (600 MHz) spectrum of R5H3 (A) and Stearyl-H3R5 (B). [file 12951_2023_1877_MOESM1_ESM.jpg]

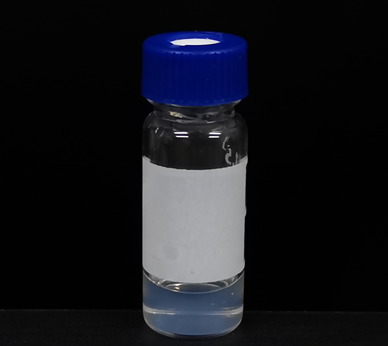

Supplement: Supplementary file 2 — Additional file 2: Figure S2. Representative image of cell-penetrating peptide modified lycorine transfersomes with components of 1,2-dioleoyl-3-trimethylammonium-propane and sodium cholate hydrate (LR@DTFs-CPP). [file 12951_2023_1877_MOESM2_ESM.tif]

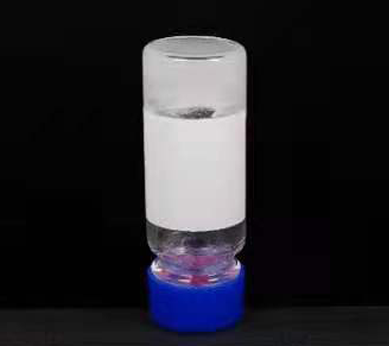

Supplement: Supplementary file 4 — Additional file 4: Figure S4. Representative image of cell-penetrating peptide modified lycorine transfersomes with components of 1,2-dioleoyl-3-trimethylammonium-propane and sodium cholate hydrate (LR@DTFs-CPP) Gel. [file 12951_2023_1877_MOESM4_ESM.tif]

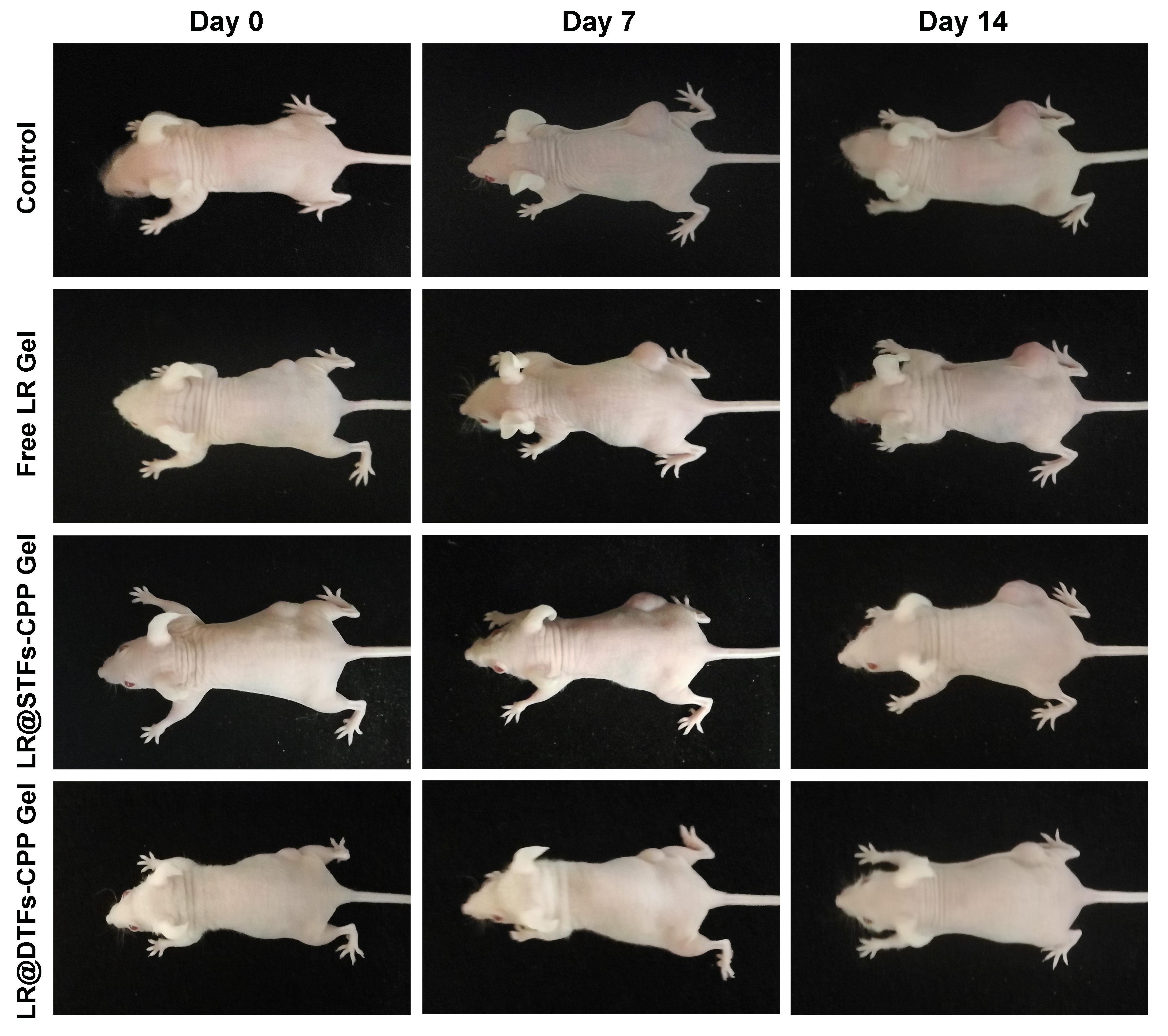

Supplement: Supplementary file 5 — Additional file 5: Figure S5. Representative images of cutaneous squamous cell carcinoma (cSCC) tumor-bearing nude mice treated with various formulations. [file 12951_2023_1877_MOESM5_ESM.jpg]

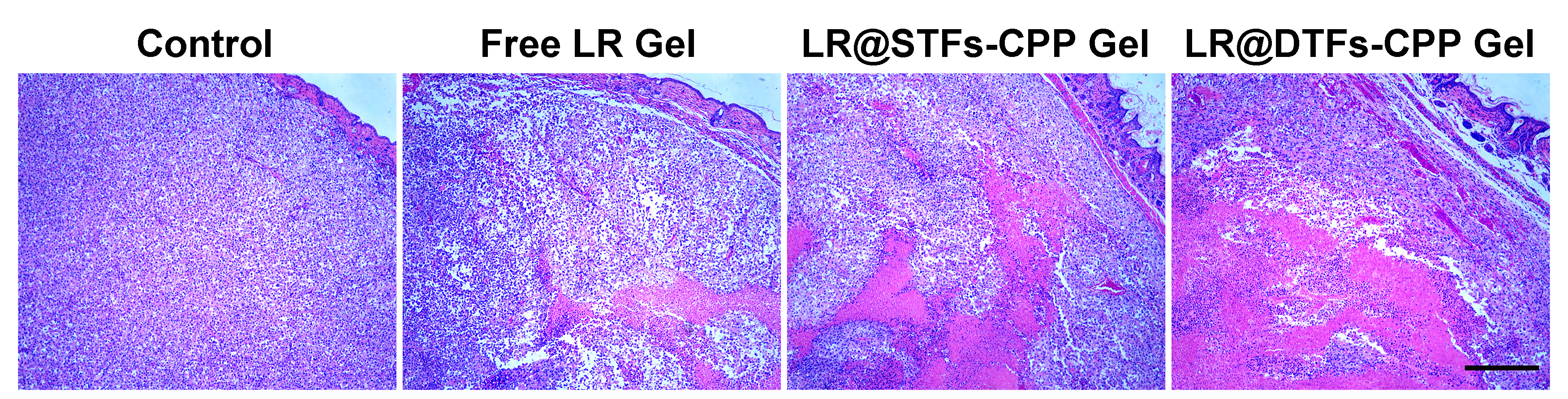

Supplement: Supplementary file 7 — Additional file 7: Figure S7. Histopathology of tissue sections of the skin with cutaneous squamous cell carcinoma (cSCC) tumor stained using hematoxylin and eosin after 14 days of treatment. Scale bar: 400 μm. [file 12951_2023_1877_MOESM7_ESM.jpg]

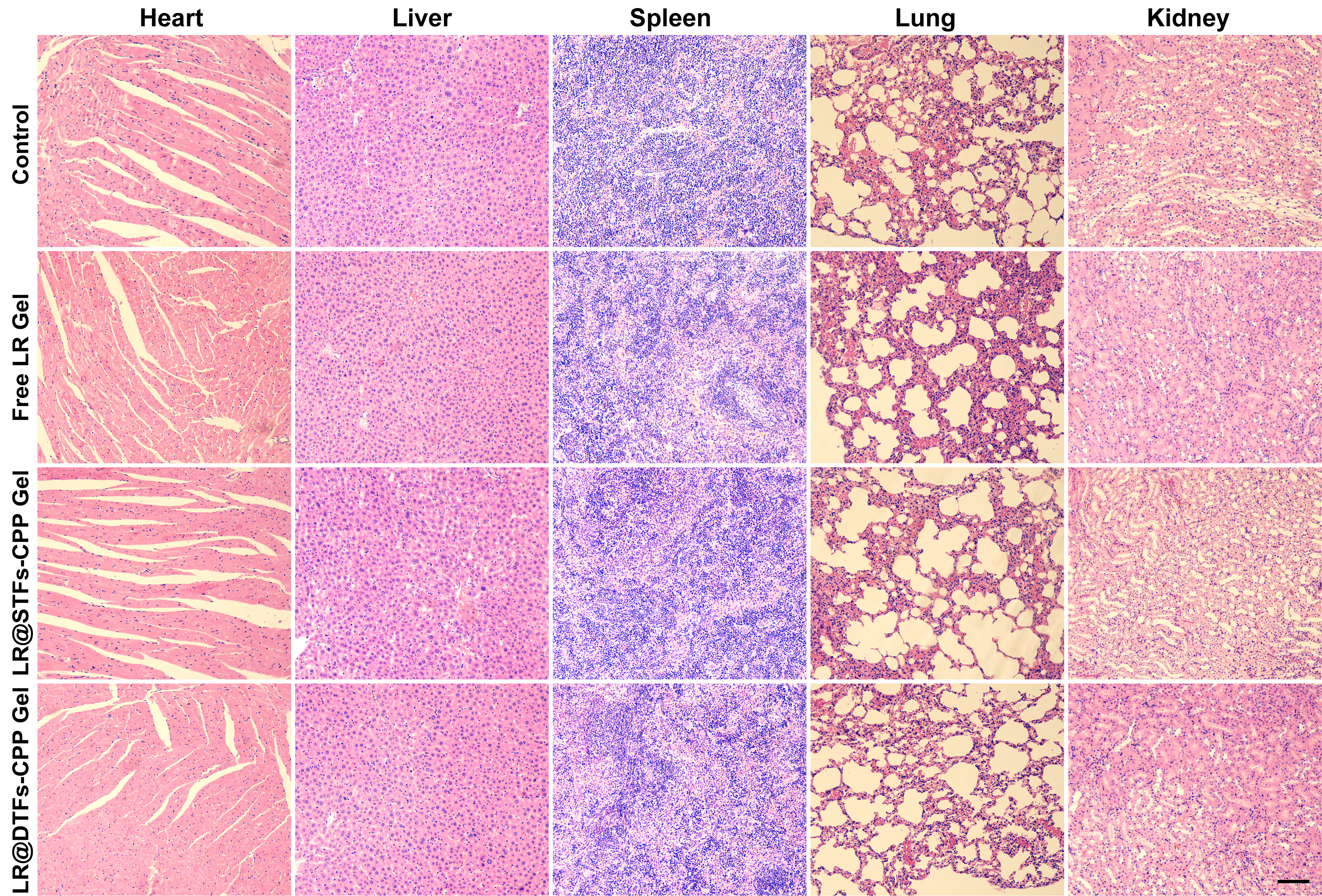

Supplement: Supplementary file 8 — Additional file 8: Figure S8. Histological assessment of the major organs of nude mice treated with various formulations for 14 days. Tissues were stained using hematoxylin and eosin (H&E). Scale bar: 100 μm. [file 12951_2023_1877_MOESM8_ESM.jpg]
